# Growing up in a rough world: scaling of frictional adhesion and morphology of the Tokay gecko (*Gekko gecko*)

**DOI:** 10.3762/bjnano.13.107

**Published:** 2022-11-09

**Authors:** Anthony J Cobos, Timothy E Higham

**Affiliations:** 1 Department of Evolution, Ecology, and Organismal Biology, University of California, Riverside, CA 92521, USA

**Keywords:** allometry, biomechanics, ecology, habitat, ontogeny, substrate

## Abstract

Many geckos have the remarkable ability to reversibly adhere to surfaces using a hierarchical system that includes both internal and external elements. The vast majority of studies have examined the performance of the adhesive system using adults and engineered materials and substrates (e.g., acrylic glass). Almost nothing is known about how the system changes with body size, nor how these changes would influence the ability to adhere to surfaces in nature. Using Tokay geckos (*Gekko gecko*), we examined the post-hatching scaling of morphology and frictional adhesive performance in animals ranging from 5 to 125 grams in body mass. We quantified setal density, setal length, and toepad area using SEM. This was then used to estimate the theoretical maximum adhesive force. We tested performance with 14 live geckos on eight surfaces ranging from extremely smooth (acrylic glass) to relatively rough (100-grit sandpaper). Surfaces were attached to a force transducer, and multiple trials were conducted for each individual. We found that setal length scaled with negatively allometry, but toepad area scaled with isometry. Setal density remained constant across the wide range in body size. The relationship between body mass and adhesive performance was generally similar across all surfaces, but rough surfaces had much lower values than smooth surfaces. The safety factor went down with body mass and with surface roughness, suggesting that smaller animals may be more likely to occupy rough substrates in their natural habitat.

## Introduction

Animals attach to surfaces in numerous ways, including claws, suction, and both wet and dry adhesion. In fact, some animals can utilize multiple attachment mechanisms [1–2], leading to multifunctionality across surfaces of varying roughness. Dry adhesion is found in many invertebrates and squamate reptiles, and has been a focus of both engineering and biological studies [3]. Models are frequently used to describe adhesion, such as the Johnson–Kendall–Roberts (JKR) model [4]. In this case, the force required to pull an elastic sphere from a flat surface is determined using the radius of the sphere and the adhesion energy between a sphere and the surface. More recent studies use the JKR model to determine the role of setal density in adhesion from insects to geckos [5]. Despite many advancements in our understanding of adhesion across organisms, few studies have incorporated ecologically relevant variables.

The ability of geckos to adhere to smooth surfaces has fascinated scientists since Aristotle, and has been followed by countless studies focused on uncovering the mechanisms of gecko adhesion, from as early as the 1800’s [6–10] to modern investigations (reviewed in [3]). Like the Lotus Effect [11], the Gecko Effect has seen a surge in attention over the past couple of decades [12]. There are over 1000 species of geckos with adhesive capabilities, with multiple origins of the system [13–14]. However, much of what is known about gecko adhesion and its associated structures is based on studies of a single species, the Tokay gecko (*Gekko gecko*) [15–19]. Additionally, the primary focus has been on adult geckos, likely given their larger size. Two key questions have received very little attention. First, how does adhesive performance vary across surfaces of different roughness? Second, how does adhesive performance and morphology vary with body size? Although the former has been the subject of a few studies, the latter has received almost no attention.

**Figure 1:**
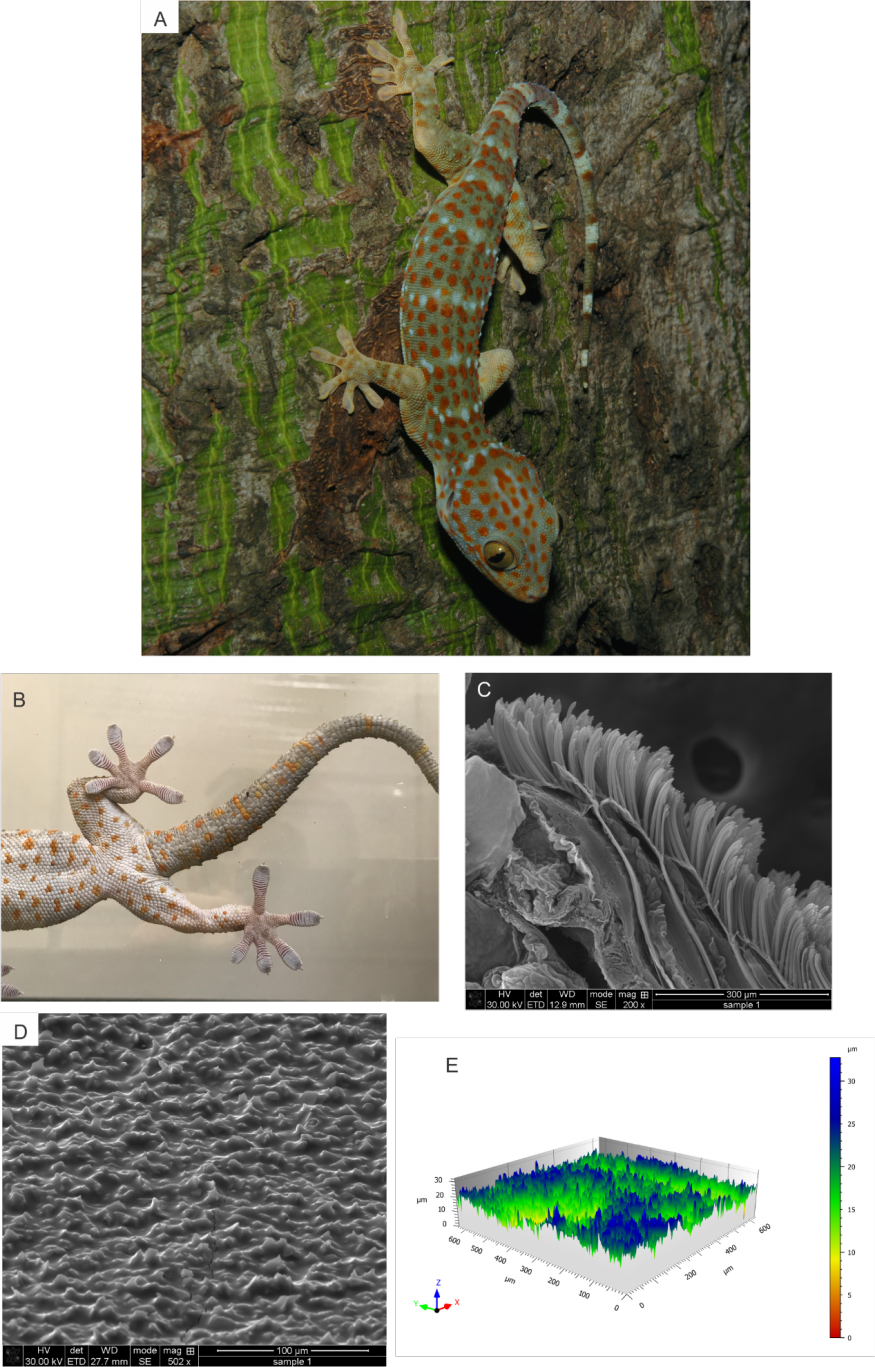
Images of a Tokay gecko in its natural habitat in Vietnam (photo courtesy of Lee Grismer. This content is not subject to CC BY 4.0.) (A), a Tokay gecko in the lab on a glass surface (photo by Timothy Higham, and has not been published previously) (B), an SEM image of the distal portion of the digit (C), an SEM image of the 2000 grit sandpaper surface (D), and a 3D image of the 2000 grit sandpaper surface using confocal laser scanning microscopy (E).

Surfaces in nature are rarely smooth and geckos are found on all types of surfaces from rough rocks to undulant tree bark [20–25] (Figure 1). Recent studies have begun to explore the role of surface roughness on frictional adhesion in geckos [1,21,25–26], and performance typically declines as roughness increases. For example, Vanhooydonck and colleagues examined the effects of substrate structure on speed and acceleration capacity in climbing geckos, and they found that acceleration was greatest on the smoothest surface (wood) where the most contact between the adhesive system and the surface could be made [27]. This illustrates that the main issue faced by geckos that are attaching via adhesive pads is the contact area between the setae and the surface. With increasingly rough surfaces, the area for contact decreases, leading to decreased adhesive performance. In a modeling framework, the force of adhesion can be related to surface energy of the substrate, the area of the adhering pad, and the compliance of the system [28]. However, most studies use widely varying surfaces [1] or uniform 3D printed surfaces [29] that do not capture the random fine-scale roughness that is likely apparent in natural habitats. For example, Huber et al. [26] measured the shear adhesive force of a single spatula on surfaces with asperities ranging from 100–300 nm, and Gillies et al. [29] manipulated the surface roughness of a macroscopic engineered rough surface in which they manipulated the wavelength and amplitude of peaks that were on the same length scale of the subdigital lamellae. Both studies found that shear adhesion was significantly reduced (up to 95% reduction of force produced on acrylic glass) on surfaces where the surface structure was close to matching the animal’s adhesive structure, highlighting the importance of considering length-scale and the impact it has on gecko adhesion when testing the effects of roughness.

Surface roughness can be qualitatively characterized in different ways (rough vs smooth), but it is a complex parameter to quantify as real surfaces in nature vary over many length scales and can have significant effects on the efficacy of an adhesive system [30–31]. At this point in time, the ability to quantify the topography of surfaces of varying roughness [21], and to replicate them [32–36], rather than using vague categorizations, allows for the possibility to test fine-scale interactions of animal adhesion and traction with more accuracy [35–37]. A key factor that is relatively unexplored is that the morphology of the adhesive system likely changes with body size, which can then impact the amount of contact made between the adhesive system and the surface on which it is clinging or moving. For example, setal length and toepad area have been found to increase with body size in the southern African gecko *Chondrodactylus bibronii* [38]. Beyond intraspecific scaling, a recent study found extreme positive allometry in toepad area among animals that have adhesive pads, from mites to geckos [39]. Thus, it is likely that animals of different size will have varying clinging and locomotor performance on rough surfaces.

Allometry plays a significant role in natural systems by imparting physical constraints of supporting different body sizes, but also in the mechanical consequences in relation to locomotion [40–43]. Scaling becomes increasingly important when structures on the surface of the animal must support the body through adhesion on vertical or near-vertical surfaces [39,44]. In the case of dry adhesives, studies have focused on the scaling of toepad morphology because of the inherent signficance for adhesive locomotion (i.e., larger toepad area leads to greater area for surface contact). In a study of geckos, skinks, and anoles, Irschick et al. [45] found a strong correlation between shear adhesive force and toepad area. However, the slope of the relationship between toepad area and body mass was lower than that of clinging ability. This suggests that there are other underlying mechanisms that contribute to clinging ability apart from pad area. Adhesive pad area across climbing taxa spans seven orders of magnitude and scales with positive allometry [44] but, after accounting for size and phylogeny, toepad area scaled with isometry or sometimes negative allometry within certain clades. A key question is how larger organisms that use adhesion will support their body weight when climbing vertical surfaces.

The scaling of adhesive components has been addressed by Webster et al. [38] and, to a lesser extent, by Delannoy [46]. In a study of *Chondrodactylus bibronii*, setal density, setal basal diamter and setal spacing did not change significantly throughout ontogeny, but pad area and setal length increased with body size [38]. Despite these increases, estimated adhesive force capacity, relative to body size, decreased with ontogeny. However, there is a mismatch between morphological measurements and measurements of adhesive force in that morphology is often used to estimate force-generating capabilities on surfaces of varying roughness. What is missing is a study that examines both the scaling of adhesion on different surfaces and the changes in morphology throughout with body size. It is predicted that increasing roughness will decrease adhesive performance due to the limited area of contact islands [21,47].

The efficacy of adhesives that mimic a gecko’s system depends upon knowing the natural interactions between the animal and the substrate. All else being equal, longer and softer setal shafts are predicted to result in better adhesion on rough surfaces due to a reduction in the effective elastic modulus [48]. However, it is currently unclear whether this translates into higher forces, relative to body mass, under whole-organism experimental studies. In order to fully understand how performance is influenced by roughness, incorporating variation in body size is important. Tokay geckos are ideal for investigating the role of body size variation given that they reach very large body sizes and they live in rainforests that likely exhibit variation in roughness (see Figure 1 for example).

Here we will measure Tokay gecko adhesive structures and compare theoretical force estimates to actual performance values to address several questions: 1) how does pad area scale with adhesive force over a significant range in body size, 2) do more compliant setae translate to higher adhesive force, and 3) do larger geckos exhibit greater adhesive force, relative to body mass? Additionally, to better understand how gecko adhesive structures interact with surface asperities on the setal level, we tested the impact of surface roughness on shear adhesion over a large size range of geckos, given that even the smallest change in setal length and compliance could impact the capacity to make local adjustments to rough surfaces [49]. To test how asperity size impacts adhesion, we generated 3D surface profiles of seven different sandpaper grits and carried out adhesion trials over a large size range of *G. gecko*. This research is significant given that most studies are limited to using 2D profiles, missing an entire axis of surface structure variation. Additionally, no study to date has quantified the effect of body size on adhesive performance.

## Results

### Scaling of morphology

**Figure 2:**
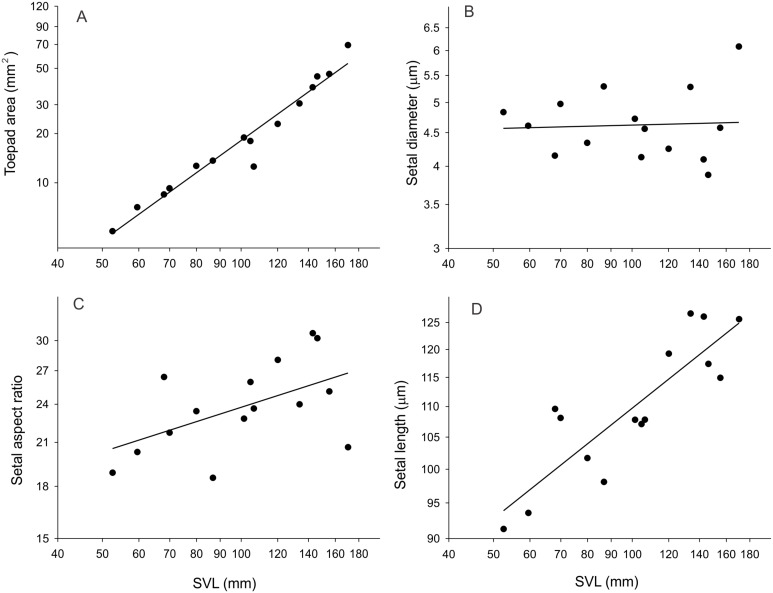
The relationship between morphological characters and SVL of 15 individuals. The relationship between toepad area (digit IV) and SVL (*y* = 2.03*x* − 6.46, *r*^2^ = 0.95) (A). The relationship between setal diameter of scansor 2 and SVL (*y* = 0.02*x* + 1.45, *r*^2^ = 0.002) (B). The relationship between setal aspect ratio and SVL (*y* = 0.2251*x* + 2.13, *r*^2^ = 0.28) (C). The relationship between setal length of scansor 2 and SVL (*y* = 0.24*x* + 3.58, *r*^2^ = 0.74) (D). Note that the axes are on a log scale and the equations of the lines are based on log–log plots for scaling purposes.

Neither density nor basal setal diameter were significantly correlated with body size (Table 1). Relative to snout-vent length (SVL), toepad area scaled with isometry (Figure 2; slope = 2.03), whereas setal length scaled with negative allometry (slope = 0.24). Using these parameters, along with the force per seta [18], estimated frictional adhesive force scaled isometrically with body mass (slope = 0.61).

**Table 1:** Scaling relationships for the variables examined in this study. Scaling is relative to SVL for morphology (first three variables) and body mass for adhesion measurements, including estimated adhesion.

variable	*N*	*R* ^2^	*P* value	exp slope	obs slope	SE slope	lower CI	upper CI	scaling

toepad area	15	0.95	<0.001	2.00	2.03	0.13	1.76	2.30	isometric
setal length	15	0.74	<0.001	1	0.24	0.04	0.157	0.328	negative
setal diameter	15	0.00	0.85	1	0.02	0.09	−0.18	0.211	NA
est. adhesion	15	0.95	<0.001	0.66	0.61	0.08	0.53	0.69	isometric
adhesion (acrylic glass)	14	0.96	<0.001	0.66	0.54	0.03	0.473	0.614	negative
adhesion (100 g)	14	0.75	<0.001	0.66	0.55	0.09	0.348	0.750	isometric
adhesion (150 g)	14	0.58	0.002	0.66	0.64	0.16	0.294	0.985	isometric
adhesion (320 g)	14	0.56	0.002	0.66	0.71	0.18	0.313	1.103	isometric
adhesion (400 g)	14	0.87	<0.001	0.66	0.76	0.08	0.576	0.938	isometric
adhesion (1000 g)	14	0.85	<0.001	0.66	0.56	0.07	0.409	0.706	isometric
adhesion (2000 g)	14	0.95	<0.001	0.66	0.60	0.04	0.509	0.689	isometric
adhesion (3000 g)	14	0.82	<0.001	0.66	0.56	0.08	0.395	0.723	isometric

### Surface roughness

The root mean square height of the sandpaper surfaces (Sq) ranged from 34.8 μm (100 grit) to 3.16 μm (3000 grit), highlighting the range of surface roughness values in our experiments (Table 2). This also encompasses much of the range of natural tree surfaces that might be found in the habitat of Tokay geckos.

**Table 2:** Roughness parameters of the surfaces used in this study. Acrylic glass is not included as it is considered perfectly smooth.

roughness measure	100 grit	150 grit	320 grit	400 grit	1000 grit	2000 grit	3000 grit

Sq (µm)	34.7876	23.9627	11.7601	16.9816	9.24755	5.13478	3.15899
Ssk	0.59911	0.58176	0.937	0.35339	0.43645	−0.05521	−0.24058
Sku	2.76697	3.84052	4.68924	2.87272	4.42821	3.43064	3.64112
Sp (µm)	103.618	93.0858	72.6184	65.311	60.2142	28.2808	17.7521
Sv (µm)	52.7945	45.2815	31.4681	48.7146	38.91	23.8727	17.4907
Sz (µm)	156.41	138.37	104.09	114.03	99.12	52.15	35.24
Sa (µm)	28.2153	18.4699	9.09496	13.7147	7.15308	4.03302	2.50632

### Frictional adhesion and safety factor

**Figure 3:**
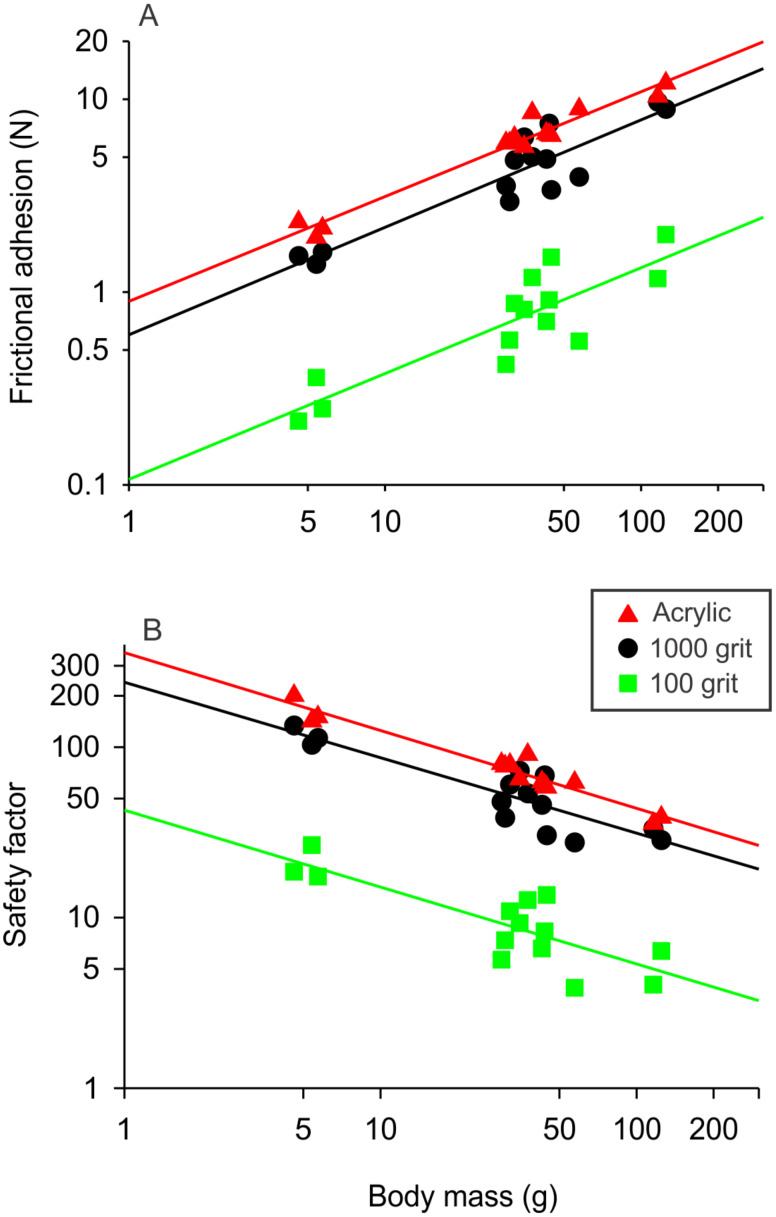
The relationships between frictional adhesion and body mass for acrylic glass (*y* = 0.54*x* – 0.05, *r*^2^ = 0.96), 1000 grit sandpaper (*y* = 0.56*x* – 0.22, *r*^2^ = 0.85), and 100 grit sandpaper (*y* = 0.55*x* – 0.97, *r*^2^ = 0.75) (A). The relationships between safety factor and body mass for acrylic glass (*y* = −0.46*x* + 2.55, *r*^2^ = 0.94), 1000 grit sandpaper mass (*y* = −0.44*x* + 2.38, *r*^2^ = 0.78), and 100 grit sandpaper mass (*y* = −0.45*x* + 1.63, *r*^2^ = 0.67) (B). Shown are values for acrylic glass (red triangles), 1000 grit sandpaper (black circles), and 100 grit sandpaper (green squares). Note that the axes are on a log scale and the equations of the lines are based on log–log plots for scaling purposes.

Larger animals generated greater amounts of frictional adhesion compared to smaller individuals (Figure 3a). In general, frictional adhesive force decreased from the smooth surface (acrylic glass) to the roughest surface (100 grit sandpaper) (Figure 3a). The strength of the correlation between body mass and adhesive force on each surface ranged from an *R*^2^ of 0.56 (320 grit) to 0.96 (acrylic glass), with the strength of the correlation generally increasing with the smoothness of the surface.

Safety factor decreased with body size and with increasing surface roughness (Figure 3b). The values ranged from 9.7 (large individual on rough surface) to 502.2 (small individual on smooth surface). Safety factor never fell below 1.0.

**Figure 4:**
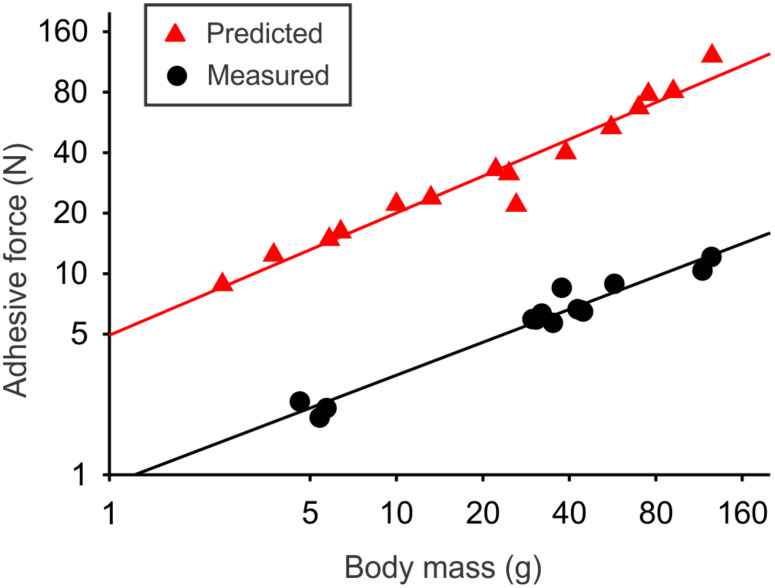
Frictional adhesive force predicted based on morphology (red triangles; *y* = 0.61*x* + 0.69, *r^2^* =0.95) and force measured experimentally (black circles; *y* = 0.54*x* – 0.05, *r*^2^ = 0.96). Note that the axes are on a log scale and the equations of the lines are based on log–log plots for scaling purposes.

When compared to the predicted force based on morphology, the experimentally measured adhesive force was significantly lower (Figure 4). However, the slopes of the log-transformed regressions were comparable.

## Discussion

Our integrative approach, combining morphology, performance, and 3D surface topography, revealed key aspects of scaling that have significant impacts on our understanding of gecko adhesion. Setal diameter and density did not change with body size, whereas toepad area, and setal length, and therefore setal aspect ratio, increased with body size. Frictional adhesion, measured experimentally, increased with body size across all surfaces. However, adhesive safety factor was not only lower on rougher surfaces, but also lower for larger animals. This has implications for ecology, especially habitat use through ontogeny, but also biomimetics. If we are attempting to mimic the adhesive system, would it be beneficial to reconstruct the adhesive system of a small gecko or a large one? Can we create an adaptable system that could achieve a constant level of adhesive performance, relative to body mass, across surfaces of varying roughness?

### Scaling of morphology and estimated frictional adhesion

Toepad area is important for gecko adhesion given than more points of contact will be made as area increases, assuming a constant density and a smooth surface. Indeed, density did not change with body mass in our study, but toepad area increased isometrically with body mass (slope of 2.03; Table 1). This is almost identical to that found for *Chondrodactylus bibronii* by Webster et al. [38]. Body mass increased relative to SVL^3.34^, which is not different from isometry. This suggests that adhesive force is greater, relative to body mass, for smaller individuals. Indeed, adhesive force estimates, based on toepad area and density, increased with body mass^0.61^. This, again, is supported by the work of Webster et al. [38]. However, this differs from the interspecific study by Irschick et al. [45], in which a positively allometric relationship between pad area and body mass was observed. As noted by Webster and colleagues [38], this is like attributable to the fact that they included many types of subdigital pad design [50]. Two explanations for our observation are outlined by Webster et al. [38]. First, the lack of a positively allometric relationship may be the result of physical constraints. Having much larger toepads, relative to body size, in larger animals would potentially lead to overlapping pads and, ultimately, disruption of adhesion. Second, smaller animals may benefit from relatively larger toepads since few contact islands are likely to be encountered in any given footfall [38]. Ecological consequences are discussed below.

### Scaling of frictional adhesion

In addition to the morphological analyses and the estimates of frictional adhesion, we experimentally measured the latter. Adhesive force increased with body size on the acrylic glass surface, with peak values approximating those in other studies. The scaling exponent was 0.54, and this was negatively allometric if we assume adhesive force is directly proportional to toepad area. This is in contrast to previous studies that examine multiple species of pad-bearing lizards that found a scaling exponent not significantly different from 1 [45,51]. One explanation for the difference between the current studies and the two former studies is that those included multiple types of toepads [50].

### Predicted versus measured frictional adhesive force

In addition to the morphological analyses and the estimates of frictional adhesion, we experimentally measured the latter. This, to our knowledge, is the first study to directly compare estimates of adhesive force (from morphology) to experimental measurements. Although the slopes and the strength of the regression are comparable between the predicted and measured frictional adhesive forces (Figure 4), there are significant differences in the actual values. The predicted forces are much higher than the measured, with the experimental values averaging 50% of the predicted values. This may not be surprising. Theoretical estimates, based on density, pad area, and the average force per seta, are reliant upon the assumption that every single seta makes contact. Our results suggest that, even on incredibly smooth surfaces such as acrylic glass, quite a few setae are not in contact with the surface. However, there are other reasons for this mismatch. The manus and pes of the gecko, when pulled across a surface, do not have all of the toes aligned parallel to the direction of movement. In a study of Tokay geckos, Stewart and Higham [16] used an apparatus to pull individuals across an acrylic glass platform while obtaining high-speed video in ventral view. They found that the average digit angle started at approximately 30 degrees and decreased to approximately 10 degrees throughout a pulling trial. At the same time, overlap among toepads within a single foot also increased, reducing the area of contact from almost 100% to approximately 75% near the end of the pulling trial [16]. The angles of the toes, never becoming completely parallel, coupled with the decreased contact area of the toepads, likely decrease the force generating capacity of the setal fields. Beyond these macroscopic factors, it is unclear whether all of the setae, in a region of the pad that is seemingly in contact with the surface, are actually engaged with the surface. There could be interactions among setae that preclude their attachment. Damage to some setae may also decrease the efficacy of setal attachment. Future work that visualizes the actual contact across the entire setal field will reveal whether this is an important factor.

### The influence of surface roughness

The roughness of the sandpaper surfaces in our study ranged from Sq = 34.8 μm (100 grit sandpaper) to Sq = 3.16 μm (3000 grit sandpaper). Acrylic glass is considered perfectly smooth. The roughness of the substrates had a large impact on the adhesive ability in Tokay geckos. Despite the similar slopes (with the exception of acrylic glass, which was negatively allometric), adhesive force on 100 grit sandpaper was, on average, only 13% of that on acrylic glass. The likely explanation for this result is that the available contact area was reduced with increasing roughness. This has been observed in other studies that model the contact between a gecko toe and surfaces that vary in asperity size [25,49,52–53]. Frictional adhesive force increased isometrically with body mass across all of the sandpaper surfaces, but this led to a decrease in safety factor (see below) since force is related to toepad area, and toepad increases at a slower rate compared to body mass.

### Ecological consequences of reduced safety factor

Safety factor (SF), as measured experimentally, decreases with body size in Tokay geckos, and it also decreases with increasing roughness. The former result aligns with the previous work on *C. bibronii* by Webster et al. [38]. They found, using estimates of frictional adhesive force, that SF decreased with body mass^−0.47^. We found that SF decreased with body mass^−0.46^ on acrylic glass and with body mass^−0.45^ on 100 grit sandpaper. Despite the similar slopes, SF on 100 grit sandpaper was, on average, only 13% of that on acrylic glass. Thus, large animals on rough surfaces will face the largest challenges due to lower safety factor. Indeed, the lowest SF was 9.7, which was a large gecko on the 100 grit surface. The largest value was 502, which was the smallest gecko on the acrylic glass surface.

What does this mean for animals in nature? We predict that these drastic differences in SF will likely dictate, to some extent, habitat use. Wright et al., 2021 examined how clinging performance in geckos and anoles on natural surfaces might predict the surfaces used in nature [24]. They found that performance on natural substrates predicted which texture (rough vs smooth) was most often used by each species. Translating this interspecific study to our intraspecific experiments, we predict that larger animals should occupy lower regions of a tree to avoid the negative consequences of falling a large distance. This is assuming that they occupy the same type of substrate as a smaller gecko. If they do fall from a high position, the impact force is likely to exceed the adhesive force capacity of their feet [54]. In terms of surface texture, they might be expected to avoid very rough surfaces in order to preserve a modest SF. In contrast, smaller geckos will have larger values of SF, and will experience lower impact forces from a fall, suggesting that they might safely occupy higher regions of a tree. Future field observations that determine the potential for ontogenetic habitat shifts are needed. Additionally, an ecomechanical model [55], incorporating contact area information from surfaces in nature, would help to understand the mechanisms underlying any shifts.

### Future directions and biomimetics

Our results detail the changes in both morphology and adhesive performance in relation to body size in a single species of gecko. These results generally align with other intraspecific studies, but not with interspecific studies. This mismatch is interesting, and requires further investigation. Specifically, there are different subdigital pad designs across geckos [50], but only one type has been investigated thus far. Do these patterns hold across geckos with different types of pads? If not, how and why?

Our results suggest important changes that occur throughout ontogeny. However, it is also clear that there are constraints in what changes are possible, such as setal width and density. These constraints may simply be due to spacing, which does not appear to change with body size. If spacing among setae does not change, it follows that the diameter of each seta is also likely to remain constant. If not, there might be negative interactions among the setal shafts (e.g., clumping). How can this be applied to biomimetics? Can we construct adaptable adhesive devices that accommodate different surfaces, or that can change depending on the need? Should robots be fitted with different systems depending on size? Although setal shaft diameter and density do not change in living animals, how does changing these conditions in artificial systems alter function? Not only might this assist with biomimetic initiatives, but it might also help us to understand why these do not occur in living animals. Regardless, we often use animals of a specific size when making connections to biomimetics, including the construction of artificial adhesives. Body size should be included given the large differences across individuals. This could be achieved by using an array of species that vary in size or, as in the case of our study, a series of individuals that span a wide range in body size. The benefits of the latter are that the general form of the toepad, and likely other aspects of the integrated adhesive system, are kept constant. However, the diversity among species will clearly offer other tantalizing insights into the possible solutions to adhesion.

## Experimental

### Morphology

We obtained 15 preserved individuals of *Gekko gecko* from local universities, encompassing the full range of body sizes for this species (mass = 4.6 g–124.9 g; snout-vent length (SVL = 52.6 mm–170.5 mm). For this part of the study, only SVL was measured given the potential issues during the preservation process. We used digital calipers (Mitutoyo Absolute) to the nearest 0.1 mm.

We used these individuals to measure the fine scale adhesive structures of *Gekko gecko*. The toe pad of the longest digit (digit IV) was first removed from the specimen and the ventral side was mounted on a glass slide using a clay putty on the dorsal side to secure the digit. A stereoscope with a mounted camera was then used to take images of each toe that were then used to measure toepad area using imageJ (version 1.53q; National Institutes of Health). Toe pad area was measured from the scansors bearing setae following previous methods [47].

Following toepad area measures, each toe was bisected sagittally and then stored in 100% ethanol for subsequent SEM imaging. The toes were removed from the ethanol and placed into a critical-point drying unit to dehydrate the tissue prior to imaging. They were then placed on a double stick conductive carbon tape affixed to a stub and sputter coated with a platinum/palladium coating. Toes were then viewed using a ThermoFisher Scientific Quanta™ 3D 200i SEM in the Central Facility for Advanced Microscopy and Microanalysis at UC Riverside (Figure 1). Each image was saved at multiple magnifications to facilitate more accurate measurements of setal length, setal diameter and setal density. Setal length was measured on the second (penultimate) scansor from the base of stalk to the tip along the midline of the shaft. Setal diameter was measured approximately 10 μm from the base of the stalk. Setal density was calculated following the methods described in [47], where setae were counted along a 32 μm length on a scansor, squared, and then multiplied by 1000 to get an estimate of setal density per mm^2^.

### Surfaces

Our experiments were carried out using a series of artificial surfaces that varied in surface topography and asperity size. In considering both the appropriate length-scale of animal adhesive structures and relevant manufactured artificial surfaces, we chose 7 different sandpapers that encompassed a range of surface roughness based on an asperity size scale from smooth to rough (as per manufacturers specifications) as well as an acrylic glass sheet. Surface asperity size ranged from ≈3 μm to ≈200 μm, well within the range of gecko setal stalks lengths (91.3 μm–125.6 μm).

The topography of each surface was visualized using a confocal laser scanning microscope (CLSM; LEXT OLS4000, Olympus Corporation, Japan), as in [32]. Mountains Map Premium version 7.4 (Digital Surf SARL, Besançon, France) was used to analyze the images obtained from the CLSM (Figure 1). Three-dimensional surface area roughness parameters were calculated from the 3D images (see Figure 1) and are presented in Table 2. Unlike 2D metrics of roughness, derived from a single transect through the sample of interest, our area roughness values stemmed from numerous surface transects. We followed the methods outlined by Higham et al. and Kumar et al. [21,32]. Although we report other values of roughness associated with 3D methods (Table 2), we focus on room mean square roughness (Sq) in this study.

### Adhesive force measurements

The specific experiments conducted in this study were approved by the University of California, Riverside (IACUC AUP 20200035). We assessed the clinging ability of 14 individuals (varying in size from hatchling to adult) on the aforementioned surfaces. These individuals were not the same as those in the morphological portion of this study, but covered a similar range in body size. The mass of each individual was obtained immediately before any performance measures were carried out to ensure that the scaling relationships were captured most accurately. We recorded mass using a standard high precision lab scale (Ohaus Scout Pro 400g). The surfaces were affixed to a portable force a gauge (Mark-10 series 5) and the animals were steadily pulled along the surface until slippage occurred or there was no more room to pull. The right manus of each individual was placed on the surface and allowed to adhere, followed by a series of 5 pulls in succession, of which the maximum force was recorded. We first determined the maximum shear adhesive force of each individual on an acrylic glass sheet to establish a control or peak performance. The remainder of the seven surfaces were then randomized for the remaining performance measures. If setae were detached from the toepads due to high forces or surface roughness issues, geckos were no longer used, and trials were resumed after the next shedding cycle or when toepads were healed. Additionally, to further attempt to achieve maximum force measurements, we tested individuals during their active periods as they are nocturnal.

Safety factor (SF) is defined as the measured performance (in Newton) divided by the force needed to support the body on a vertical surface. The latter was calculated as body mass multiplied by acceleration due to gravity. Our measurements on single appendages were multiplied by 4 and then divided by the force required to support the body. Values of 1 indicate that the adhesive force is equal to the force required to support the body. Values less than 1 indicate that the animal can no longer hold its body on a surface.

### Statistics

We used linear models (LM) using the package ‘stats’ in Rstudio (version 4.1.2; Rstudio, Inc., USA) to determine how morphological traits scaled with SVL, and how adhesion scaled with body mass. We then used the package ‘ggplot2’ and generated regression plots of each model. Scaling was assessed by first log10-transforming each variable in order to linearize the data. We then obtained the slope of the regression and compared that to the expected values of isometry. For example, for linear measurements, a slope of 1 (relative to SVL) or 0.33 (relative to body mass) indicates isometry. The 95% confidence intervals around the slope were used to determine the allometric relationships.

## Supporting Information

File 1 Adhesion data.

File 2 Morphology data.
